# A Rare Case of Double-Headed Psoas Minor Muscle with Review of its Known Variants

**DOI:** 10.7759/cureus.1312

**Published:** 2017-06-05

**Authors:** Matthew Protas, Vlad Voin, Joy MH Wang, Joe Iwanaga, Marios Loukas, R. Shane Tubbs

**Affiliations:** 1 Department of Anatomical Sciences, St. George's University School of Medicine, Grenada, West Indies; 2 Research Fellow, Seattle Science Foundation; 3 Seattle Science Foundation; 4 Department of Anatomical Sciences, St. George's University School of Medicine, Grenada, West Indies; 5 Neurosurgery, Seattle Science Foundation

**Keywords:** congenital anomaly, posterior abdominal wall, anatomy, anatomic variation

## Abstract

Variations of the muscles of humans are important to remember for those who interpret imaging and for those who operate near these anomalies. Herein, we describe a rare two-headed psoas minor muscle found incidentally during dissection of the posterior abdominal wall. This case is presented with a detailed review of all known variations of the minor psoas and is analyzed through a literature review.

## Introduction

The psoas minor muscle (PMM) is a flat, small, and fusiform shape muscle innervated by the first lumbar nerve that runs ventromedial to the surface of the psoas major muscle. Its function is to aid in the flexion of the lumbar spine, stabilization of the hip joint and to act as a tensor of the iliac fascia. Unilateral flexion of the PMM causes the lumbar spine to tilt sideways [1]. The PMM has a small, muscular part (7.85 cm), which is then continuous with a long tendon (13.13cm) [2]. Guerra, et al. determined that the tendon comprised 57% of the total length [2]. The PMM originates from the 12th thoracic vertebra, first lumbar vertebra, and the corresponding intervertebral disc. The muscle descends inferiorly where it becomes a long tendon that inserts onto the pectineal line of the pubis, iliopectineal eminence and laterally into the iliac fascia [1, 3]. Of all the muscles in the body, the PMM is well documented as having the greatest propensity for agenesis (56%), which varies with race but not sex [3]. When present, the muscle has been noted to have variable sites of origin, insertion, as well as sexual and racial dimorphisms. Detailed literature present on human variations of the PMM is scarce and ambiguous. During a routine cadaveric dissection, we identified a cadaver found to have a double-headed PMM. This phenomenon has only been reported four other times in the extant literature and is the rarest of all PMM variations. The last description in English was in 1875 by Macalister [1, 3-4]. The aim of this study is to shed light on this extremely rare variation as well as to perform a review of all variation of the PMM found in the literature to aid in better understanding of the clinical applications of these variations for surgeons, radiologists, and embryologists. Informed consent statement was obtained for this study.

## Case presentation

**Figure 1:**
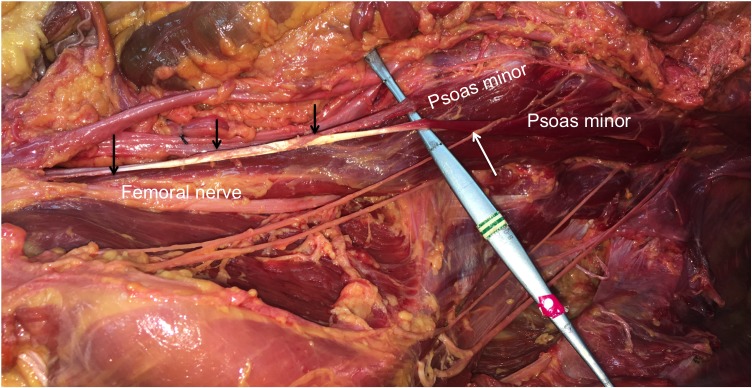
Dissection: two-headed psoas minor The left posterior abdominal wall as dissected in this case. Note the two heads of the psoas minor and the relationship to the genitofemoral nerve is shown (white arrow). The distal tendon of the psoas minor is also highlighted (black arrows)

A 49-year-old male cadaver at death with a history of pancreatic cancer underwent routine dissection of the posterior abdominal wall. On the left side, a two-headed psoas minor was found (Fig. 1). The lateral head arose from the lumbar L1 vertebral body and the medial head originated from the L4/L5 vertebral bodies and intervening intervertebral disc. The genitofemoral nerve exited the psoas major between the two heads of the psoas minor but nearer to the medial edge of the lateral head. The lateral head was innervated by the genitofemoral nerve but a clear branch to the psoas minor was not identified. The two heads of the psoas minor came together at the lower spinal segment (S1) vertebral level and continued as a single tendon to attach onto the iliopectineal eminence. No other anatomical variations were noted in this specimen.

A review was conducted using PubMed, Excerpta Medica Database (Embase), Scopus, Cochrane Library, and Google Books and Google Scholar to retrieve studies reporting variations of the psoas minor muscle; it was last updated in January 2017. The medical terms and text words used were “psoas minor muscle + variations” or “psoas minor muscle + morphology” or “psoas Parvus muscle + variations” or “embryology of the psoas muscle”. Appropriate publications in the reference lists of the most relevant articles were also manually searched. There were no restrictions on the dates of publications reviewed. Exclusion criteria were papers not published in English nor Spanish. Inclusion criteria required a confirmed diagnosis of a variation of the psoas minor muscle.

## Discussion

### Variations of origin

Common variations of the PMM are related to the spinal level of origin. Typically, the PMM originates from thoracic vertebrae (T12), L1, and the intervertebral disc between them. It has also been seen to arise from only L1 or L2 and the intervertebral disc [3-4]. The PMM can also originate from the subdiaphragmatic fascia and medial arcuate ligament [5]. Muscle fibers have even been seen originating from the crus of the diaphragm [5]. Of all the variations of the PMM having two heads as seen in our case illustration, is the rarest and least described. Its prevalence, exact origin, measurements, or images are completely absent from the available literature. In 1732, during a cadaveric dissection, Winslow first described the double head psoas minor with the second head lying deep to the first [3]. Our case differs in that, the two heads were found to be side by side and not one lying deep to the other (Fig 1). Since then, Macalister, Kelch, and Cruvelhier described that it may split partially or completely prior to its insertion [3-4]. Of the four manuscripts to describe its appearance in depth, Macalister is the only one in English [4]. This variation of the psoas minor has been reported to insert on the lesser trochanter in conjunction with the psoas major [1, 4, 6]. One of the major problems with literature regarding this variation is that it has been consistently improperly cited. 

### Variations in the insertion

When bifurcation of the psoas minor tendon is noted, the aberrant band inserts onto the synchondrosis between the fifth lumbar vertebra, iliopectineal line, and sacrum [1, 4]. The insertion of the tendon can also be continuous with the pelvic fascia or directly with the iliac fascia [1, 4]. Guerra, et al. described variations where the tendon can insert into the pectineal line of the femur, neck of the femur, lesser trochanter with the iliopsoas, the arched line, the iliac fascia, the inguinal ligament, or the pectineal ligament [2]. Guerra, et al. and Macalister described variation in four fetuses where the PMM passed posteriorly to the crural arch and inserted onto the pectineal line of the femur [2, 4]. In Ojha, et al.’s analysis of 30 cadavers, they reported five cases where the muscle belly was thick and the tendon insertion was short and broad and attached to the iliopectineal eminence and pecten pubis [7]. They also reported three cases of a thin-bellied PMM with a tendon that was long and fanned. The tendon inserted near the iliopectineal eminence and merged with the obturator fascia medially and the iliac fascia laterally.

Psoas accessories are a frequent variation that was described in 1889 by Clarkson and Rainy as muscular fibers from under the surface of the tendon of the psoas minor passing downward to fuse with the internal surfaces of the iliacus and psoas major muscles [8]. During an examination of 22 fetuses, Guerra, et al. described fanlike expansions coming from the tendon of the PMM that joined the fascia of the psoas major muscle [2]. This suggests that psoas accessories could indeed be due to an incomplete separation between the psoas major and minor muscles during embryogenesis. Garg, et al. described a variation where the deep fibers of the PMM muscle and superficial fibers of the psoas major muscle can be interdigitated [5]. They, however, did not report information on the prevalence or where the attachments were specifically, making it unclear if these were psoas accessories.

### Agenesis

The most common variation of the PMM is its agenesis. Of the five muscles that commonly undergo agenesis, the PMM is the most common (pyramidalis, peroneus tertius, palmaris longus, and plantaris). The accepted prevalence of the PMM is between 33.4 and 52% [1]. In a 450 cadaveric study, Anson reported its absence 183 times bilaterally and 69 times unilaterally [7]. Bergman, et al. reported agenesis bilaterally in 50% of cases. In a study of 2,627 cadavers, bilateral agenesis occurred 54.5% of the time [3]. In an 182 subject study, 70 had bilateral PMM, 12 had right-sided, and eight were left-sided [3, 7]. In a 30 cadaver study, the prevalence of the PMM was only 26.66% [7]. Garg, et al. reported the presence of the PMM to be 36.67% of which 81.81% were bilateral and 18.19% were unilateral [5]. A fetal study revealed that of the 22 fetuses, the PMM was present in 13 (59.09%) with 10 of them bilateral and three unilateral [2]. In a 500 cadaveric study, Seib reported a PMM prevalence of 38.6% [9]. The study also concluded that the muscle was more frequently absent in females. Contrary to that notion, Bergman, et al. noted that it was more frequently absent in males [3]. The current medical consensus is that there is no difference in prevalence of the PMM between sexes [4, 6]. Garg, et al. in his examination of 30 cadavers determined in females, the PPM was thinner, narrower, and had a longer tendinous insertion than males [5].

Racial disparity of the presence of the PMM has been well described. Hanson, et al. discovered a huge disparity in PMM agenesis when comparing young white (13% agenesis) and black (91% agenesis) males [10]. In another study, the prevalence of agenesis in Asian subjects was 49.9% (361 cadavers), 57% (2203.5 cadavers) in white subjects, and 66.6% in (337 cadavers) black subjects [3]. Loth's examination of the racial disparity of agenesis of the PMM revealed the rate to be: Russians (48%), Alsatians (57%), English (59%), Scotsmen (63%), Irish, (66%), Blacks (52.4%), and Chinese (51.9%) [9]. Hanson, et al. described that morphologically, the PMM when present in whites was thicker compared to blacks [10].

### Clinical applications

The major clinical implications of the PMM is that of psoas minor syndrome and its ability to spread infection and malignancy to the retroperitoneal region of the body. Psoas minor syndrome presents as pain in the iliac fossa due to increased tension of the PMM. Pain is exacerbated by palpation of the taut tendon. The symptoms appear due to compression of retroperitoneal neurovascular structures [7]. Symptoms from psoas minor syndrome can also mimic that of diverticulitis and appendicitis and needs to be ruled out. The accepted treatment is a tenotomy [7]. Patients with psoas minor syndrome will have difficulty performing various exercises, especially jumping. It is unknown and needs to be further investigated if variations are the cause or whether certain variations increase the propensity for patients to develop psoas minor syndrome. A similar investigation should be conducted on whether these variations can increase the rate and propensity for infection and malignancy to spread to the retroperitoneal region. Garg, et al.’s describes the variations of PMM where it originates from the diaphragmatic fascia and medial arcuate ligament, as well as another where fibers of the muscular belly of the psoas minor originated from the crus of the diaphragm, can lead to these infections and malignancies spreading to the endothoracic cavity [5].

## Conclusions

In this paper, we present a rare case of a two-headed psoas minor muscle found incidentally during dissection of the posterior abdominal wall. Our analysis of available literature produced a thorough review of all known variants of the psoas minor. Further studies are needed to fully elucidate the effects of these psoas minor variants on clinical function and pathologies.
